# Review: Computational analysis of human skeletal remains in ancient DNA and forensic genetics

**DOI:** 10.1016/j.isci.2023.108066

**Published:** 2023-10-04

**Authors:** Ainash Childebayeva, Elena I. Zavala

**Affiliations:** 1 Department of Archaeogenetics, Max Planck Institute for Evolutionary Anthropology, Leipzig, Germany; 2 Department of Anthropology, University of Kansas, Lawrence, KS, USA; 3 Department of Molecular and Cell Biology, University of California, Berkeley, Berkeley, CA, USA; 4 Department of Biology, University of Oregon, Eugene, OR, USA

**Keywords:** Molecular biology, Computational bioinformatics, Paleogenetics, Archeology

## Abstract

Degraded DNA is used to answer questions in the fields of ancient DNA (aDNA) and forensic genetics. While aDNA studies typically center around human evolution and past history, and forensic genetics is often more concerned with identifying a specific individual, scientists in both fields face similar challenges. The overlap in source material has prompted periodic discussions and studies on the advantages of collaboration between fields toward mutually beneficial methodological advancements. However, most have been centered around wet laboratory methods (sampling, DNA extraction, library preparation, etc.). In this review, we focus on the computational side of the analytical workflow. We discuss limitations and considerations to consider when working with degraded DNA. We hope this review provides a framework to researchers new to computational workflows for how to think about analyzing highly degraded DNA and prompts an increase of collaboration between the forensic genetics and aDNA fields.

## Graphical abstract

**Figure:**
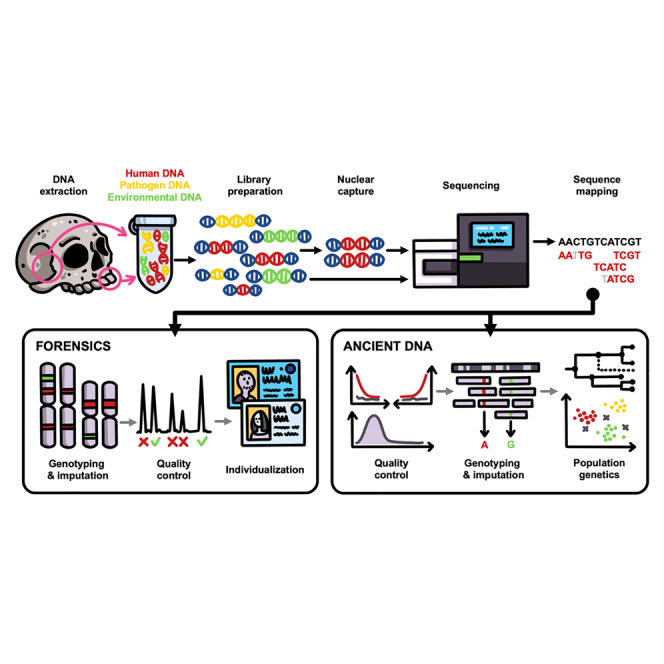

## Introduction

Human genetics is a cornerstone of both the fields of forensic genetics and ancient DNA (aDNA). In forensic genetics, this typically relates to linking DNA recovered from a piece of evidence to a specific individual. This can include not only matching DNA profiles, but also information about an individual’s phenotype,^1^ genetic ancestry,^2^ and/or relatives^3^ that may be paired with non-genetic evidence to narrow the investigative space. In aDNA, recovered DNA has been used to learn more about past human interactions, kinship structures and migrations,^4^^,^^5^^,^^6^^,^^7^ test evolutionary hypotheses,^8^ and to study phylogenetic relationships between archaic lineages and their modern representatives.^9^^,^^10^^,^^11^ Degraded DNA is a hallmark of aDNA, due to the time periods of the remains from which data are generated. Human identification (HID) casework, a subset of forensic genetics that includes disaster victim identification, active and cold cases, and historical identifications, also deals with degraded DNA depending on the time periods and environmental conditions of the recovered human remains.^12^^,^^13^^,^^14^ The challenges faced with generating DNA profiles from degraded DNA are therefore shared between the forensic genetics and aDNA fields. Overlaps and benefits of exchanging laboratory protocols between these fields have been previously outlined.^15^^,^^16^ In this review, we build on this foundation by focusing on the impacts of degraded DNA on the computational portion of analysis while highlighting overlapping and distinct features between forensic genetics and aDNA.

The key characteristics of degraded DNA are its relatively short fragment length (30–70 base pairs), limited quantity, and damage patterns,^17^^,^^18^^,^^19^^,^^20^ each of which presents a challenge that both fields have worked to overcome for data generation and analysis. Conventional laboratory methods for isolating non-degraded DNA from different sources (DNA extraction) and preparing it for downstream analysis favor the exclusion of small DNA fragments, which are typically thought to be artifacts or uninformative. These protocols have thus needed to be altered for application to degraded DNA. Early exchanges of DNA extraction protocols between the fields^21^^,^^22^^,^^23^^,^^24^ have continued through the decades, leading to the recovery of DNA fragments less than 50 base pairs^14^^,^^25^^,^^26^^,^^27^^,^^28^^,^^29^ and establishing pre-treatment protocols for contamination removal.^30^^,^^31^^,^^32^ The later due to the low endogenous DNA content of degraded samples, which makes them susceptible to exogenous contamination from other DNA sources (i.e., microbial and non-degraded human DNA). This has resulted in all pre-DNA amplification steps being carried out in specialized clean room laboratories dedicated to aDNA work,^20^^,^^33^^,^^34^ with similar guidelines being established for forensic analyses.^35^

Degraded DNA is typically fragmented and present in low quantities which is a challenge for preparing the extracted DNA for downstream analyses. DNA cloning was used to identify the first aDNA fragments,^36^^,^^37^ but this method often generated artifacts that led to false positives. While the advent of PCR helped to overcome this challenge, damage patterns and short fragment sizes resulted in low amplification efficiency and the co-amplification of often indistinguishable contaminant DNA.^19^^,^^20^ The advent of next-generation sequencing (NGS) technology has provided an avenue for data generation through parallel sequencing of millions of DNA molecules and downstream bioinformatic processing. As with the initial data generation steps, the analysis of NGS data has required the development of bioinformatic tools and techniques to address the difficulties arising from the degraded nature of the DNA source, which is the focus of this review.

**Figure 1:**
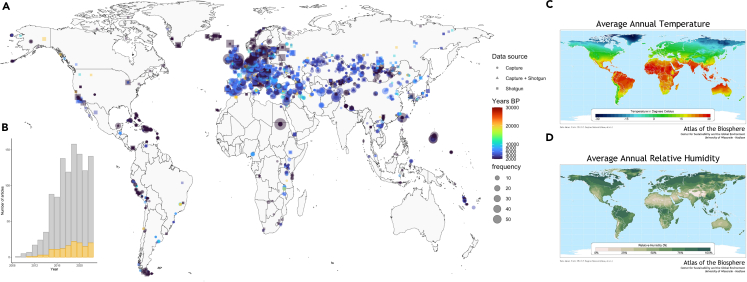
Distribution of published ancient DNA data and frequency of forensic genetics NGS studies (A) Map of published aDNA data. Frequency indicates a number of individuals, Years BP = thousands of years before present, Data source = capture data, shotgun data, or a combination of both. AADR v54.1^63^ was used for metadata; (B) A histogram of the number of articles with titles, abstracts, or keywords that include forensics and mention NGS or massively parallel sequencing (gray) and degraded (yellow) based on a search on scopus.com; (C) Average Annual Temperature map (The Nelson Institute Center for Sustainability and the Global Environment, University of Wisconsin-Madison); (D) Average Annual Relative Humidity map (The Nelson Institute Center for Sustainability and the Global Environment, University of Wisconsin-Madison).

Despite all these challenges, in the last decade, the publication of more than 10,000 genome-wide and whole-genome data from ancient humans has made it possible to learn more about human evolutionary history and genetics, even in areas that are known to be challenging for DNA preservations due to high ambient temperatures and humidity (Figures 1A, 1C, and 1D). NGS-based methodologies have expanded the information that can be gained from degraded DNA samples beyond the traditional forensic DNA profile standard of short tandem repeats (STRs).^38^^,^^39^^,^^40^^,^^41^^,^^42^ Although STRs are unlikely to be replaced for routine forensic casework due to their prevalence in existing databases and the ease of generating STR profiles, NGS analysis of SNPs has gained traction. SNPs have been shown to be more effective for generating DNA profiles from degraded remains,^43^^,^^44^^,^^45^^,^^46^^,^^47^ including enabling the identification of individuals through more distant relatives (investigative genetic genealogy, IGG) instead of via first-generation relatives or a direct match.^48^^,^^49^ The growing interest for NGS in forensic studies is exemplified by the marked increase in studies related to NGS in forensics in the last decade (Figure 1B). The increase in the number of laboratories performing research on degraded DNA, publications of step-by-step laboratory protocols,^26^^,^^50^^,^^51^^,^^52^^,^^53^^,^^54^^,^^55^ computational pipelines,^56^^,^^57^^,^^58^ and workflow primers^55^ has helped to ensure transparency and reproducibility of processing between aDNA datasets. Within forensics, organizations such as the Scientific Working Group on DNA Analysis Methods (SWGDAM)^59^ (in the US), the European Network of Forensic Science Institutes (ENSFI),^60^ and the International Society of Forensic Genetics (ISFG)^61^ have served as platforms for discussion, sharing of protocols, and the development of guidelines for quality assurance of forensic DNA analysis. However, in both fields, step-by-step pipelines of the computational workflows, including discussions around limitations, are limited.

Since the advent of NGS, a natural widening has occurred between the forensic and aDNA fields. The legal connotations of forensic casework and its consequences on people living today require forensic laboratories to adhere to strict quality assurance standards for laboratory accreditation and strict IT requirements.^62^ This includes performing verification and validation studies before the implementation of new wet laboratory methods and software (including any version updates). These rigorous standards necessarily slow the integration of new technology into forensic genetics practice, emphasizing the importance of understanding the limits and factors impacting the accuracy of new methods and techniques. Leveraging the flexibility of the aDNA field to explore and test new methods has the potential to narrow the search space for advancing forensic genetics technology, as has already been discussed for laboratory methods.^15^ In this review, we focus on the computational workflows performed in forensic genetics and aDNA analysis when working with low-coverage NGS data. This includes a discussion of limitations and contextualizing the decision-making processes involved at different steps. We hope this both provides a solid foundation for those new to computational analysis in either field, but also prompts interdisciplinary conversations that will lead to mutually beneficial advancements in forensics and aDNA.

## Sampling and laboratory work

**Figure 2:**
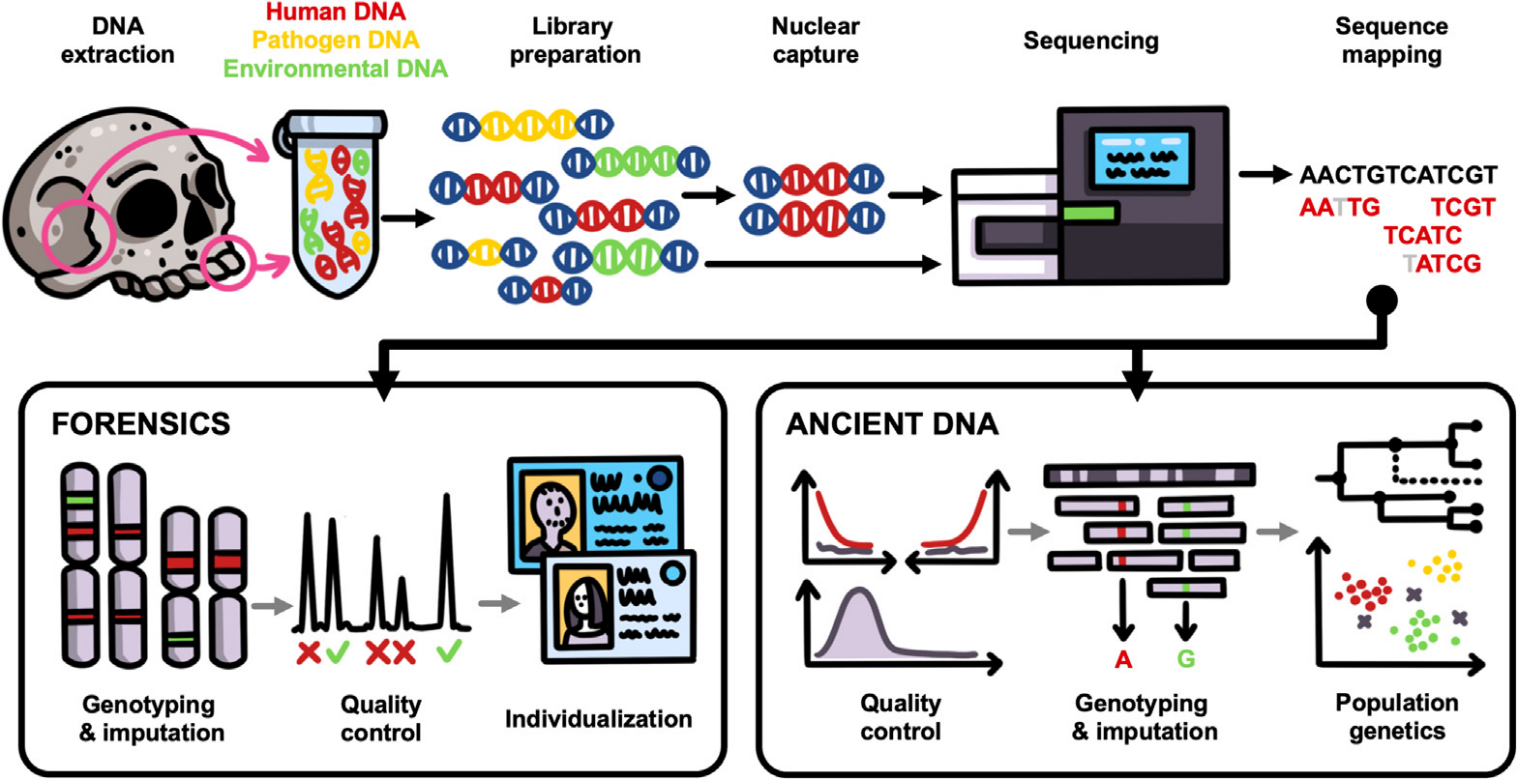
Simplified workflow for forensic and ancient genetic analyses The first step in the workflow is DNA extraction in a dedicated pre-PCR lab room. Following the DNA extraction, a genetic library is constructed by adding barcodes/indices and adapters to the DNA for downstream sequencing. The library is then sequenced for downstream bioinformatic analyses. In some cases, targeted capture is done to enrich for specific targets or types of DNA, also followed by NGS and downstream analyses.

The general laboratory workflow for degraded DNA analysis can be divided into five steps: sample preparation, DNA extraction, library preparation, in some cases targeted enrichment, and sequencing (Figure 2). The genetic material used for the analyses covered in this review is typically recovered from skeletal material; however, rootless hair has also been shown to yield degraded DNA,^64^^,^^65^^,^^66^ including in commercial forensics applications.^67^ DNA is extracted and purified from bone or tooth powder that has been drilled or ground from a particular skeletal element. The resulting DNA extract contains all DNA extracted from the sample, including microbial DNA and other non-endogenous DNA (contamination) from individuals or animals who may have come into contact with the remains of interest. Extracted DNA is then converted into libraries by adding adapter sequences, which allow it to be sequenced. Included in this library preparation step is the addition of indices (short oligos) to the DNA library molecule, which work as a tag to identify which DNA sequences are associated with which library. Depending on the data quality and types of questions that are being addressed, enrichment of specific loci may also be performed prior to sequencing. While this provides a brief overview, there are different considerations that must be made for each step.

Due to the limited availability of ancient remains, the importance of these remains for relatives and descendant communities, or the historical and biological record, many studies have focused on reducing the amount of bone material used, while maximizing the amount of DNA recovered.^68^^,^^69^^,^^70^ While the petrous portion of the temporal bone has been found to be a rich source of DNA in both forensic and aDNA studies,^71^ due to its importance for understanding hominin evolution and associated invasive sampling requirements, teeth and other long bones are often substituted depending on the sample quality and availability.^70^ For the recovery of highly degraded DNA (<100 base pairs in length), different versions of an inorganic DNA extraction method^26^^,^^72^^,^^73^^,^^74^ are used in both aDNA and forensics, paired with either *double- or single-stranded (ss) DNA library preparation*.^53^^,^^75^^,^^76^^,^^77^^,^^78^ Library preparation for forensics is typically amplicon based and sequencing directly follows the completion of this step. For “younger” (<∼30,000 years) aDNA samples that have relatively better quality DNA, the double-stranded library preparation method is typically coupled with a partial uracil-DNA glycosylase (*UDG*) *treatment* to repair DNA damage while preserving the deamination on the terminal ends to allow for aDNA authentication.^79^ Decisions around library preparation methods and whether whole-genome shotgun or targeted enrichment is performed impact how the data are analyzed downstream as will be discussed in the following section.

**Figure 3:**
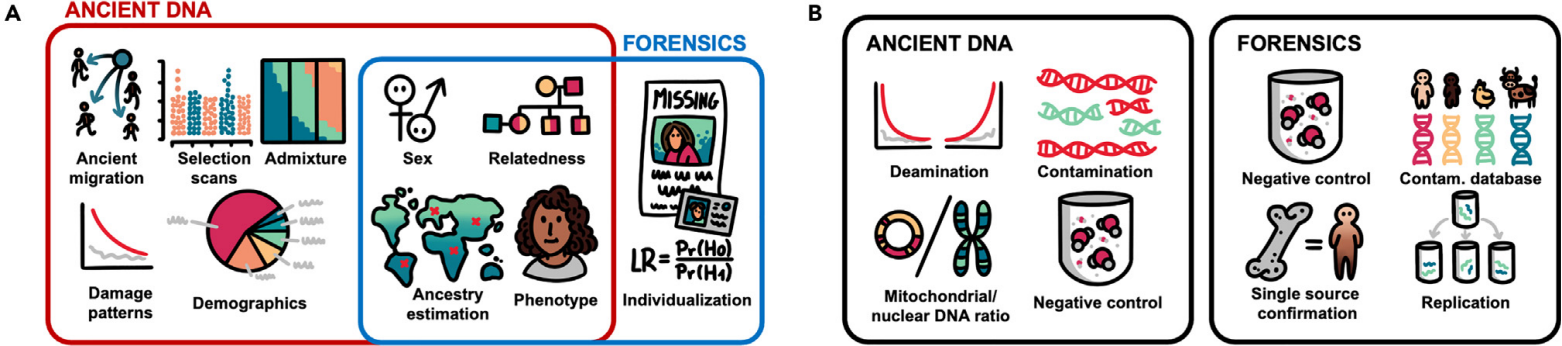
Comparison of aDNA and forensic genetics analyses and quality control measures (A) The different types of conventional downstream analysis currently performed in aDNA and forensic genetics fields as well as (B) quality control measures for monitoring contamination and confirming endogenous DNA.

Specific regions or types of DNA are sometimes targeted for enrichment (e.g., hybridization capture) in order to decrease sequencing costs and/or restrict the type of genetic data that is produced.^44^^,^^80^^,^^81^^,^^82^ Which regions of the genome are targeted for analysis differ between forensic and aDNA analyses. Forensic studies typically focus on common SNPs that are informative for individualization and may also include SNPs that provide insights about genetic ancestry, phenotype (skin, hair, and eye color), or can be used for identifying genetic relatives^44^^,^^49^^,^^83^(Figure 3A). Concerns around genetic privacy of individuals whose data may be collected for forensic databases also motivates the use of SNP panels instead of whole-genome sequencing (WGS) in forensic casework to minimize collecting medically informative data.^84^ Ancient data capture arrays target SNPs that are informative for evaluating genetic variation on a population rather than individual scale. One array commonly used in aDNA studies is the “1240k” SNP capture array that targets ∼1.2 million single-nucleotide positions representing global genetic variation, as well as functional SNPs and SNPs under selection,^4^^,^^85^ which has been commercially available since 2021.^86^ An updated version of the array, known as the “Twist Ancient DNA” assay,^87^ is able to enrich for ∼1.4 million SNPs, containing additional SNPs not present on the 1240k array. A larger set of ∼3.7 million SNPs adds additional SNP panels to the 1240k set that are informative for genetic variation observed in Neanderthals and Denisovans.^85^ In addition, the aDNA field generally promotes open data sharing.^88^ However, there are cases when open data sharing is discouraged, for example, when working with Indigenous groups^89^ (see the Public Databases section for further discussion).

## Initial bioinformatic processing

**Figure 4:**
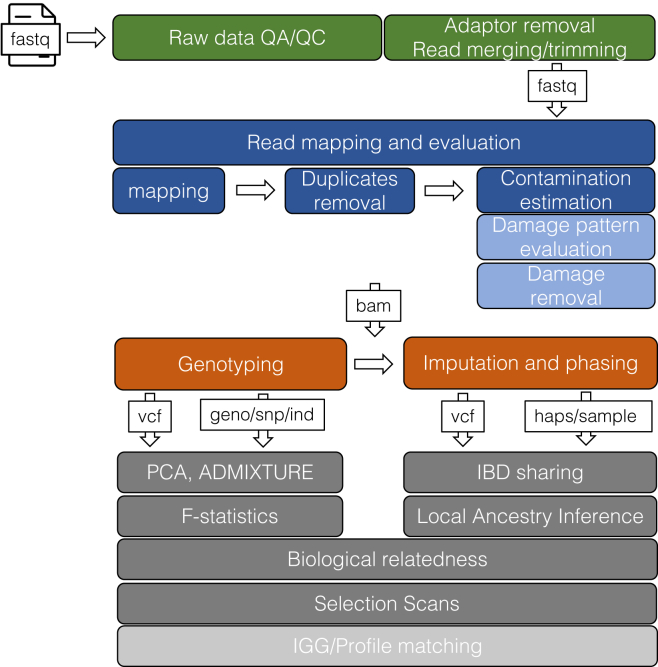
General bioinformatic processing steps in aDNA and forensic genetics Light blue color indicates steps that are more relevant for aDNA, while light gray for forensics.

In order to perform downstream analysis, raw sequence data must undergo preliminary bioinformatic processing (Figure 4). This generally includes demultiplexing (assigning sequences to their specific libraries based on their assigned indices), trimming of adapter sequences, removal of PCR duplicates, filtering based on length and quality metrics, and mapping of sequences to a reference genome. These preliminary steps are performed in both fields. Subsequent NGS forensic genetics analysis can be split into three categories (amplicon-based sequencing, enrichment or capture, and WGS) all of which are typically performed with commercial kits. Amplicon-based sequencing is widely used within forensics as it helps to maintain similarity to previous amplicon-based genetic workflows, compatibility with existing databases, and is required for correctly identifying STR alleles due to challenges in determining the start and end positions of these short repetitive regions. Unless unique molecular identifiers are used, PCR duplicate removal is not performed for amplicon-based sequencing. For mtDNA analysis, different commercial software packages are available that have specifically been designed to work with mapping mtDNA (a circular reference) and improved calling of indels (e.g., QIAGEN’s CLC Genomics Workbench,^44^^,^^90^ AQME,^91^ and SoftGenetics’s GeneMarker HTS^92^) that also allow users to visualize the *pileup* of reads. Nuclear DNA kits are often paired with commercial bioinformatic workflows that take in sequencing data, perform demultiplexing, adapter trimming, and mapping, and then present the user with genotype calls (e.g., Verogen’s FGx system,^93^ or Thermo Fisher Scientific’s HID Ion GeneStudio S5 System^94^^,^^95^). The development of bioinformatics workflows for sequencing applications in forensics is rare as forensic laboratories may not have the personnel (bioinformaticians) or the flexibility to develop such pipelines when adhering to the information management and IT standards of government data security systems. Thus, commercial software packages such as Parabon Fχ Forensic Analysis Software Platform, QIAGEN’s CLC Genomics Workbench, and SoftGenetic’s NextGENe may be used for analysis of WGS and capture data, each of which includes a deduplication step in addition to the adapter trimming and mapping. While helpful for reproducibility, it is often difficult for the general public to directly test how different data qualities impact specific workflow elements via simulations or other testing as these workflows are typically not open source.

Initial bioinformatic analysis of aDNA data is conventionally performed as follows. After demultiplexing, raw sequencing FASTQ files are trimmed to remove sequencing adapters,^96^ and the reads are mapped to the reference genome using tools like bwa^97^ or Bowtie^98^ with relaxed parameters^99^ to produce BAM files. Duplicate reads are removed using tools such as Picard MarkDuplicates^100^ or Dedup.^101^ The aDNA status is then authenticated based on deamination, represented by C-to-T substitutions on the 5′ end (and G-to-A substitutions on the 3′ end in double-stranded libraries) (see Deamination in Figure 3B), and using tools like MapDamage2.0^102^ or DamageProfiler,^103^ which allow the user to both visualize and quantify the damage patterns in the data. Based on the damage profile, read trimming is performed to remove the DNA damage, which accumulates at the terminal ends of the reads, with the number of the bases trimmed depending on the type and architecture of the library preparation. Two to three bases can be trimmed from a double-stranded UDG-half library,^104^ eight to ten bases from a double-stranded non-UDG library (assessed per library), while the double-stranded full-UDG libraries are not trimmed. In the case of non-UDG ssDNA libraries, the number of bases trimmed is typically performed on the terminal bases in a library-dependent manner.

### Identifying contamination

Contamination in the context of genetic analysis of an individual’s skeletal remains refers to the presence of any DNA that is not from that individual. The presence of contamination is a concern for both forensic and aDNA analyses and each field has developed different techniques for monitoring its presence (Figure 3B). Forensic laboratories maintain databases of DNA profiles from potential contamination sources (individuals who are involved in casework, have been in the laboratory, etc.) and previous casework which can be compared to genotyped casework samples.^35^ Internal validation studies are used to set coverage thresholds for calling consensus alleles and determining that a profile is predominantly from a single individual.^105^^,^^106^ This is also part of what drives the high-coverage thresholds in forensic analysis. Data that are determined to represent multiple individuals are excluded from downstream analyses. Finally, in some forensics laboratories, when possible at least two skeletal elements or bone powder aliquots per individual are processed and the DNA profiles between these extracts are required to match.^12^ This independent replication of results also serves as a means to check for concordance between replicates, and to monitor for sample switches that may have occurred during batched downstream processing. Both forensic and aDNA fields carry contamination controls, such as no-template controls also known as negative controls and reagent blanks containing all reagents used in the experiment minus the sample,^107^ from DNA extraction and library preparation steps through to sequencing to identify potential contamination from reagents or handling in the laboratory.

Methods for estimating contamination in aDNA studies are often based on the haploid DNA elements and rely on measuring heterozygosity levels: X chromosome in males,^108^^,^^109^ or mtDNA (contamMix^110^^,^^111^ and schmutzi^112^) in both males and females. Other methods include comparing the contamination in reads with and without aDNA damage,^113^ or evaluating the breakdown of linkage disequilibrium.^114^ Unambiguous sex determination can also be used as a metric for contamination assessment, except in instances of same-sex contamination, as well as the unambiguous determination of mitochondrial and Y chromosome haplogroups. The methods listed previously that are based on confirming the presence of a single individual can also be used in HID casework as exemplified in a recent study on the analysis of hair from Ludwig van Beethoven.^115^

#### Limitations of contamination metrics

Each of the contamination evaluation methods described previously has its own limitations, the impacts of which are dependent on the data that are being evaluated. Methods based on the coverage of the X chromosome are most effective when applied to male individuals for detecting female contamination. Detecting male contamination is still possible, but requires determining if there are multiple X chromosomes resulting in polymorphic loci. Methods that rely on mtDNA overcome this issue, but still require at least 3-fold coverage depth (e.g., schmutzi^112^) and an even coverage of the mitogenome. Other methods were designed for specific sample preparation and sequencing parameters (e.g., AuthentiCT^113^), such as single-stranded library preparation and paired-end sequencing. More generally, contamination estimation methods tend to work more reliably on higher coverage data, and are not as accurate when used on low-coverage samples. Users must consider which tools are most applicable to their data and may decide to use multiple methods. It should also be noted that contamination estimates for the mtDNA and nuclear DNA can differ and it is therefore necessary to monitor the mt/nuclear (nc) DNA ratio.^116^ The mt/nc ratio is known to vary between and within the same bone sample,^117^ and can influence the contamination estimates by underestimating nDNA contamination when extrapolated from mtDNA in cases of high mt/nc ratio.^116^ Overall, for aDNA analysis, a contamination level of 5% is often considered an upper threshold for inclusion in the downstream analyses.^109^^,^^114^

### Chromosomal sex determination

Sex determination based on evidence for the presence of different sex chromosomes is common practice in forensic and aDNA analysis for both inferring biological sex and evaluating the presence of contamination. Within the forensic field, sex determination is sometimes performed by looking for the number of copies of the amelogenin gene where two copies indicate a male and one indicates a female. However, this test is not always reliable as a deletion of this gene in males has been observed,^118^^,^^119^ leading to the use of other regions of the Y chromosome.^120^^,^^121^

In aDNA studies, different methods for chromosomal sex determination are used based on the data analyzed. The first and perhaps most straightforward approach is to evaluate the ratio of average coverage across the X and Y chromosomes compared to the average coverage across autosomal chromosomes,^122^ while normalizing for the target size of each chromosome.^123^ The expectation for this method (after normalization) is that males with one X chromosome would have half the coverage on the X compared to females. This method has been shown to work with at least 1,000 reads, but should only be applied to WGS data. When using capture data, different methods are used to correct for the preferential enrichment of regions of the autosomal and sex chromosomes which impacts expectations around coverage ratios. When limiting analysis to a set of “390k” array SNPs (a subset of the 1240k SNP panel), a ratio of reads mapping to the Y and X chromosomes is calculated as Y/(Y + X).^4^ When expanding the analysis to the full 1240k SNP panel, these ratios are corrected by the number of bases targeted on each chromosome.^124^ Another technique, which has been used for both low-coverage WGS and capture data, is to calculate the ratio of the average coverage of the X chromosome to the average coverage of X and autosomal chromosomes (X/(X + auto)).^125^ Care should be taken when applying this technique to capture data, which is already known to not work as expected with the 1240k SNP panel. Sanity checks by comparing calculations with similarly processed data for individuals of known chromosomal sex can be helpful in these situations. All methods mentioned will be impacted by the presence of contamination and also are created to differentiate between a binary where XX and XY are the two possible outcomes. Alternative methods have been developed for identifying other karyotypes, which have resulted in the identification of ancient individuals who may have had Klinefelter syndrome (XXY).^126^^,^^127^

### Summary statistics

The points for making decisions around data quality and downstream processing differ between the forensic genetics and aDNA fields. In forensic workflows, qPCR is performed on DNA extracts to quantify the amount (nanograms) of DNA present in the extract and to detect potential PCR inhibition.^128^^,^^129^^,^^130^ This step is used for calculating input volumes for library preparation. Evaluation of contamination and coverage estimates is then combined with the genotyping and analysis steps. The motivation behind this workflow is the large numbers of samples (predominantly non-degraded), time constraints, and that validated workflows cannot be changed in order to enable direct comparisons across laboratories and to maintain laboratory accreditations.

In contrast, many aDNA laboratories perform low-coverage WGS to evaluate the data quality and make decisions around how, and if, to generate more data. In studies with large numbers of individuals that are presumed to have DNA of similar quality, a subset of skeletal elements may be evaluated for their DNA preservation before making decisions that are applied to the full set of skeletal remains. The set of summary statistics used for this initial data quality evaluation typically include the percent of sequences that map to the reference genome (in total and for a certain length cutoff), duplication rate, deamination percentages, coverage of the reference genome, complexity of each library, and contamination. The **percent of mapped reads** informs on how much human DNA (endogenous and contamination) is present in a DNA library relative to all sequences (and categorized by a minimum length, typically 25–35 base pairs). If a library contains a high percentage of short DNA fragments, gel cuts, or physical separation of DNA band(s) above a certain fragment length from an agarose gel for downstream analysis, may aid in decreasing sequencing costs.^53^ As this is a time-intensive and complex protocol, it is not recommended for routine use. **Duplication rates** can be a reflection of the amount of unique DNA molecules in a library, since the more times that the same original DNA molecule is sequenced the less likely it is that new, unsequenced molecules are still present in a library. High duplication rates can indicate that increased sequencing depth will unlikely result in an increased genome coverage. **Deamination rates** are used to determine the aDNA status, and an arbitrary cutoff of at least 10% observed C-to-T substitutions on terminal ends is used to indicate the presence of aDNA in non-UDG-treated libraries, or C-to-T substitutions in only the two terminal bases of reads in UDG-half libraries. **Coverage** estimates begin to provide information about how much data are sequenced from a given library; however, this is only informative for the portion of the library that was sequenced. To determine how much data may still be available in the library, we recommend using **complexity estimates**. This can be measured by first calculating the number of informative sequences present in the library (percentage of mapped reads above a certain length and quality threshold multiplied by the number of molecules present in the library as determined by qPCR).^131^ The number of informative sequences are then multiplied by the average fragment length of filtered reads and divided by the genome target size (i.e., 3 billion for the human genome). This metric provides an estimate for the theoretical coverage that can be obtained from a library if every DNA molecule is sequenced. Alternatively, library complexity can be calculated bioinformatically after sequencing using tools like Picard (GATK). **Contamination** estimates (discussed previously) will provide information as to what percentage of the previously calculated complexity is endogenous DNA. These numbers can then be used to estimate the cost and feasibility of generating different genome coverages. It is at this point where decisions are made as to whether to proceed with data generation and if WGS or SNP capture-based approaches should be pursued. While WGS is the gold standard when it comes to the amount of data generated and the potential analyses available, SNP capture is a more cost-effective approach when taking into account the low endogenous content of the aDNA data.

## Genotyping

### Forensic genetics

The term genotyping, while focused on allele determination at specific loci, has been used to refer to different segments of workflows from sample preparation to allele determination using various methods (e.g., capillary electrophoresis). In this review, we will refer to genotyping as the process by which sequencing data are used to determine alleles at specific loci.

Software coupled with amplicon-based NGS kits for forensic applications use a binary or threshold genotyping approach where the sequencing read pileup at each position is examined to determine if a locus is homozygous or heterozygous. Genotype calls are based on predetermined analytical thresholds for allelic coverage and heterozygous balance and typically require relatively high coverages at each locus (e.g., >650 reads for amplicon-based sequencing^93^). For degraded samples profiled with NGS, a 10X reporting threshold has been used for both mtDNA^12^ and SNPs.^48^ Validation studies are key for setting reporting thresholds as outlined in SWGDAM guidelines,^132^ FBI Quality Assurance Standards,^133^ and ENSFI best practices manuals.^134^^,^^135^

Probabilistic genotyping offers an alternative to the binary approach that can include models evaluating multiple factors (e.g., number of individuals, heterozygosity, amount of data) and incorporate prior knowledge based on available reference data (e.g., sequencing error, allele frequency errors, patterns of linkage disequilibrium),^136^ to provide a probability that each genotyped SNP is correct. Genotype probabilities can then be incorporated into downstream analyses and allow for more informed decisions on which SNPs to include in a final DNA profile. The benefits of integrating probabilistic genotyping methods into forensic genetics have been recognized via validation studies and guidelines.^137^^,^^138^ While most of these studies are focused on STR and mixture analysis, the application of probabilistic genotyping to degraded, single-source samples has in recent years begun to be explored for both identification of human remains and for identifying potential perpetrators in criminal cases.^45^^,^^139^ Notably, the probabilistic genotyping method used in the study by Gordan et al., 2022^45^ (ATLAS^140^) was developed for aDNA data and allows the user to take into account deamination rates, which have been shown to be present in historical remains.^14^ Due to the highly degraded quality of DNA in ancient studies, it is unsurprising that methods from this field may be useful for forensic casework involving historical and/or degraded remains.

### Ancient DNA

Genotyping of aDNA is often split into two categories depending on the data quality and planned downstream analyses. The first is pseudo-haploid genotyping, which involves randomly selecting a single read per position in place of calling a true diploid haplotype. This is typically performed when working with low-coverage data, genome-wide array data, or when analyzing a large number of genomes where the majority are low coverage. There are different available software for performing pseudo-haploid genotyping, including pileupCaller and bam-caller (Table S1). Each of these softwares allows users to specify which SNPs should be genotyped and allows filtering based on coverage, mapping quality, and base quality. They can either randomly select a read from the pileup or, given sufficient coverage, select the allele supported by the majority of the reads. This step should be performed after additional end trimming to minimize impacts of deamination. For non-UDG or partial-UDG ssDNA libraries, deamination or contamination impacts can be further minimized by limiting calls of C-to-T SNPs to the reverse strands, and G-to-A to the forward strands only. In addition, after genotyping, one can quantify the observed number of transitions and transversions (C>T, A>G, A>C, etc.) to determine if this ratio (also known as Ti/Tv) differs from the expected value of 2–2.1 for WGS data.^141^ However, this method will not work for capture arrays where the Ti/Tv ratios significantly deviate from the expectation.

Probabilistic genotyping is generally used for aDNA samples with better coverage. There are different software that can be used to determine genotype likelihoods in ancient samples: snpAD,^142^ ATLAS,^143^ bcftools,^144^ GATK,^145^ ANGSD,^108^ and others (Table S1). A set of reference genotypes from modern data is often employed to aid genotyping ancient samples, a commonly used one being the 1000 Genomes reference dataset.^146^ Again, trimming of termini is important prior to diploid genotype calling. However, tools like ATLAS^143^ are able to take into account the aDNA damage when determining genotype likelihoods and thus additional preprocessing is not necessary prior to the genotyping. Moreover, when using non-UDG data, genotyping can be restricted to transversions only, which are not prone to aDNA deamination, and are thus more reliable for downstream analyses, as well as restricting to damaged reads with PMDtools.^147^ Non-UDG ssDNA library data further allow for processing reads separately, which can serve as an additional control.

#### Limitations and considerations

Limitations of genotyping can be examined from two different perspectives: genotype accuracy (error rate and allelic dropout) and the impact of this accuracy on downstream analyses. Here, we focus on the former for currently used methods in forensic and aDNA work based on evaluations with simulation studies, which allow decoupling of laboratory and bioinformatic parameters. This excludes analysis pipelines paired with commercial kits.

Low coverage is a known concern for genotyping as it can result in allelic dropout and increased stochasticity in allele sampling, complicating differentiation between heterozygous and homozygous loci. A recent forensic case solving a 16-year-old double murder in Sweden^48^ encouraged pairing WGS with SNP panels as a check for these issues. Conventional forensic genotyping also does not take into account the presence of damage patterns, which have been observed in DNA recovered from historical and forensic casework.^14^^,^^148^^,^^149^^,^^150^ Due to the high-coverage values required for typical forensic casework, low rates of damage are negligible and are not expected to impact downstream analyses, but may have an impact on low-coverage samples. Practices from the aDNA field of trimming ends of reads to remove damage patterns or utilizing probabilistic genotypers that take postmortem damage into account may open up more degraded samples for HID analysis.

Genotyping of low-coverage aDNA data with pseudo-haploid calling in theory does not have a limit as, given coverage by at least one read, an allele can always be selected. However, the presence of contamination can decrease the chance of randomly sampling reads from an endogenous content. In parallel processing, limiting analyses to putatively deaminated fragments can serve as a sanity check for evaluating if certain signals are contamination driven. Another check is to use the f4-statistic, a summary statistic measuring correlations in allele frequencies between four populations^151^ (see the downstream analyses section for explanation of f statistics), in the form f4 (all fragments, deaminated fragments; set of test modern populations, outgroup). If there is no contamination, the resulting statistic should be ∼0, i.e., indistinguishable from 0. Reference bias is also a concern and can be checked with an f4-statistic if there is a diploid version of the genotype as well (i.e., in scenarios where higher and low coverage data are co-analyzed). The f4-statistic can be used to detect reference bias in pseudo-haploid testing, when set in the form f4 (diploid genotypes, pseudo-haploid genotypes; reference genome, outgroup). A significant negative f4-statistic would indicate attraction between the pseudo-haploid data and the reference (reference bias). In case of archaic individuals, pseudo-haploid data may be attracted to the outgroup via the so-called “long-branch” attraction.

Reference bias continues to be a concern for probabilistic genotyping, which is relevant for both modern and ancient applications. This bias was identified when researchers discovered that higher genotype probabilities are assigned to calls that are homozygous with respect to the reference used for alignment, which are composed predominantly of European and African ancestry.^152^ Evaluating reference bias for genotyping individuals from underrepresented populations continues to be assessed^153^ and many studies focused on these groups start with an evaluation of genotyping accuracy for identifying rare variants. Modern reference databases may not fully represent the genetic variation of individuals involved in forensic casework or past populations studied in aDNA. Moreover, modern individuals represent already admixed states, and thus exhibit different patterns on linkage disequilibrium and potentially shorter haplotypes compared to the more ancient un-admixed sources. When deciding which methods to use, it is important to remember that different degrees of uncertainty can be tolerated for genotyping in forensics and aDNA. In the case of aDNA, more relaxed quality control metrics, and reliance on population-wide estimates allows for usage of lower quality and quantity data compared to the forensic data where the goal is identification of individuals. The potential implications of making an error are also significantly greater in forensics where genetic evidence is used in court cases. However, there is also potential for aDNA to directly impact present-day people, for example aDNA evidence can be used by Native American tribes to gain U.S. federal recognition.^154^

## Imputation

Imputation, or filling in missing genotype information, is a common procedure in both modern and ancient datasets that allows the inclusion of additional genetic information based on patterns of linkage disequilibrium across the genome.^155^^,^^156^ Reference datasets provide information on what alleles are more likely to be inherited together. Imputation methods often involve a phasing step where maternal and paternal chromosomes are separated into haplotypes (for review, see the study by De Marino et al.^157^). Phasing often relies on using a reference dataset, or related trios (parents and offspring), as short read sequencing is not informative on the background of alleles. Common uses for data after imputation and phasing include identity-by-descent (IBD) calling, local ancestry inference, selection scans, demographic modeling, and other analyses. When performing imputation, reference panels of worldwide populations are regularly used (such as the 1000 Genomes Reference panel^146^); however, in cases with large numbers of test samples, the use of a reference set may not be necessary.^158^

### Forensic genetics

Imputation is just beginning to be explored for forensic applications with low-coverage data.^48^^,^^159^ Direct-to-consumer testing companies, such as FamilyTree,^160^ and GEDmatch,^161^ have databases that have been used for IGG and typically type between 0.7 and 1.6 million SNPs. Commercial forensic sequencing labs like Astrea Forensics use imputation in their pipeline for recovery of low-quality DNA for comparison to direct-to-consumer tests (Astrea Forensics, California, USA). Imputation has the potential to increase the ease of comparing DNA profiles generated from these different platforms and also improve chances of identification from low-quality samples with partial DNA profiles by producing a more complete profile. It could even allow for matching between STR and SNP profiles.^162^ However, as the reference panels used for imputation are predominantly derived from populations of European ancestry, questions have been raised about the accuracy of using them for inferring SNP genotypes for individuals from underrepresented populations.^163^ A recent study evaluated (1) the accuracy of two imputation programs (Beagle^164^ and Gencove) and (2) the impact of using currently available reference panels for samples from different African populations.^153^ It was found that, at 4X coverage for the five African populations included in the study, 38% of common variants and ∼50% of rare variants could not be imputed, likely due to variance in genetic distance to the reference panels.^153^ Continued studies are needed to explore potential biases introduced by available reference panels when applied to diverse populations for individualization purposes.

### Ancient DNA

Common imputation software used in aDNA studies includes Beagle,^164^ GeneImp,^165^ and GLIMPSE.^166^ Generally, imputation starts with producing genotype likelihoods. These likelihoods are then used together with a panel of modern high-coverage populations to determine the most likely genotypes based on linkage disequilibrium patterns observed in the reference data.^167^ The DNA coverage cutoff of 0.5X is often used as the inclusion criterion for imputation, since samples with lower coverage are less likely to produce accurate genotype calls after imputation.^168^^,^^169^^,^^170^ In the future, greater availability of high-coverage WGS ancient genomes may be able to overcome this issue by generating curated high-quality reference datasets of ancient individuals only, and thus removing the need for modern reference datasets.

Although powerful, there are important limitations to consider when applying imputation to degraded DNA. These include damage patterns, contamination, and if one is using WGS or capture data. WGS data allow for imputation of a greater number of positions in the genome and are less prone to ascertainment bias. With capture data, it is not possible to detect new variation or private variation present in a population not used in the ascertainment. Using modern reference panels contains the same concerns as those outlined in the genotyping section. In addition, due to the nature of the imputation procedure, the homozygous reference genotypes are more likely to get high imputation scores, while homozygote alternative and heterozygous genotypes often have lower post-imputation accuracies, which can result in a reference bias after imputation.^168^ For ancient individuals, this limits the types of populations that can be successfully imputed. Despite this limitation, a recent study found that with 1X coverage imputation can result in >99% genotype concordance with a minor allele frequency threshold of 0.1^171^ with Beagle v4.0.^172^ The accuracy of imputation can be assessed by downsampling high-coverage ancient or modern DNA data.^170^ When evaluating imputation accuracy, it is important to remember that aDNA coverage and damage are often non-randomly distributed, and thus a randomly downsampled genome does not necessarily represent the true low-coverage state.

A general consideration when using imputation both in forensics and aDNA is the quality of the reference dataset. Worldwide genetic variation is not represented equally in publicly available reference datasets and thus comparison of imputed samples from various parts of the world should be done with caution.^173^

## Downstream analyses

### General population genetics analyses

Ancestry-informative markers are commonly included in forensic sequencing kits with the aim of providing investigative leads and/or aiding in improved accuracy for downstream population genetics analyses. The goal of such work is to evaluate the ancestry of an individual. Although conceptually different, the term ancestry is often used interchangeably with race and ethnicity in various fields, including medical genetics, pharmacogenetics, forensics, and others.^174^^,^^175^ Thus, guidelines around the communication of this information to law enforcement that decouple genetic ancestry from race and ethnicity, particularly as categories differ country by country and through time, is an area of active discussion and attention in the field due to concerns around interpretations and dissemination of results.^176^^,^^177^

Principal component analysis (PCA) and ADMIXTURE^178^ or STRUCTURE^179^ analyses are among the most common population genetics methods that are performed on modern and aDNA to better understand the population structure and broader genetic affinity among individuals and populations. Projection PCA, wherein ancient samples are projected upon modern genetic variation, is commonly used to overcome the low coverage and the presence of damage patterns in aDNA, and the SmartPCA software from EIGENSOFT v7.2.1 (http://www.hsph.harvard.edu/alkes-price/software/) is often used for this purpose.^151^ Briefly, ancient samples are merged with modern worldwide populations, and a set of populations is chosen as a reference set upon which all other samples are projected. Multidimensional scaling (MDS) is another dimension-reduction method that has been used to assess broad relationships between sets of individuals in a hypothesis-free manner, similar to PCA. Ancient individuals with as low as 1% endogenous DNA have been assigned correctly to their geographic origin using MDS.^180^ It is important to note that population genetics analyses like PCA and MDS are most informative when used in concert with other methods like F-statistics, ADMIXTURE, and reference population sets. ADMIXTURE^178^ analysis is used to cluster individuals based on their genetic ancestry. In an ADMIXTURE analysis, ancient samples can be analyzed using a modern reference, or without one if a sufficiently large number of individuals from relevant population sources are available. Based on the admixture analysis and PCA, genetic ancestry and admixture components are often formally tested using qpAdm and F-statistics.^151^^,^^181^

### Biological relatedness

The identification of relatives is of interest in both forensic and aDNA studies. In forensic human identification casework, in the absence of a direct match in available databases, kinship analysis with STRs (familial searching) has been used to identify potential perpetrators of violent crimes, unknown remains, or determine paternity. False negative and false positive rates of this analysis have been evaluated and found to be impacted by likelihood ratio cutoffs use, the type of relationship in question, and the individual’s genetic ancestry.^182^^,^^183^^,^^184^ One of the commonly used software for this analysis, Familias,^185^^,^^186^ uses allele sharing between genetic relatives that are identical by descent (IBD) to identify first- to second-degree relatives.

To identify more distant genetic relatives, IGG utilizes SNPs to either calculate total shared segments of DNA (measured in centiMorgans, cM) or by computing kinship coefficients based on allele sharing and pairwise differences. There are several informative, in-depth reviews and studies on this approach and its application in forensic settings.^187^^,^^188^^,^^189^^,^^190^ For the first category, a threshold is used to differentiate between segments that are identical by state and IBD.^189^ The total number of shared cM used for IGG relationship estimates is largely based on tests using European populations for relatively close familial relationships (1st to 3rd degree). The frequency with which more distant genetic relatives are misidentified as closer genetic relatives across various population backgrounds based on the number of shared cM is unknown. The second category relies on reference panels to determine allele frequencies and to control for potential population substructure. This approach is commonly used in the medical genetics and aDNA fields (as described in the following section) and has the benefit of working on smaller subsets of SNPs, as it is not dependent on identifying IBD tracks.^189^ Another category estimates IBD tracks between individuals and therefore does not rely on allelic frequencies.^191^^,^^192^^,^^193^ Verogen has recently leveraged the likelihood approach to develop their ForenSeq Kintelligence kit, which contains 10,230 SNPs identified as being maximally informative for identifying genetic relatives.^194^ The limitations of these methods are still being explored. A recent study found that while incomplete SNP profiles (>50%) had a minimal impact on relative identification, 1%–5% of genotyping error resulted in reduced accuracy for the segment-based identification methods often used in IGG.^195^

The certainty of individual identification in forensic casework is often based on a likelihood ratio that expresses how likely a certain DNA profile would be observed from the individual in question versus from a random person in a specific population. This calculation utilizes allele frequency data from select populations to determine likelihood ratios per population. SWGDAM guidelines indicate how to report and describe these ratios.^196^

Methods used to assess biological relatedness among aDNA samples include pairwise mismatch rate (PMR), ancIBD,^197^ READ,^198^ and lcMLkin.^199^ PMR, lcMLkin, and READ provide pairwise relatedness information, while ancIBD^197^ can be used to find links between more distant relatives based on the IBD sharing. PMR and READ can be used on genotype calls, while lcMLkin relies on genotype likelihoods, and ancIBD is based on imputed and phased data. Most methods that are used to determine biological relatedness in aDNA data are only able to determine biological kinship up to a second degree. Some, such as PMR and READ, do not separate between parent-offspring (PO) and full siblings (FS), while others, lcMLkin and ancIBD, can be used to differentiate PO from FS, and identify more distant relatives.

More recent methods that have been developed to determine biological relatedness among individuals rely on imputed and phased data. One of these methods is ancIBD, which assesses pairwise haplotype sharing in a set of samples. IBD-based methods can generally differentiate between PO and FS and identify more distant relatedness, such as 4-5th degree, as well as avuncular relatedness in some cases. Generally, a combination of several methods to estimate biological relatedness is used. Additionally, uniparental marker data (Y- and mtDNA haplotypes), age at death, and archaeological context are used when building family trees. There are important caveats and limitations to consider when estimating relatedness, such as consanguineous relationships, increased background relatedness due to a population bottleneck, and sample coverage (lower coverage may increase relatedness). Determining mtDNA haplotypes and heteroplasmy for low-quality data, including deconvoluting mixtures, is another area of overlap between aDNA and forensic genetics.^40^^,^^149^^,^^200^^,^^201^^,^^202^^,^^203^

### Admixture and genetic introgression

#### *F-statistics*

F-statistics are a commonly used suite of methods in aDNA to test for various scenarios of admixture and population relationships.^151^^,^^204^ In forensics, F-statistics have been used for quality control and STR evaluation and analysis,^205^^,^^206^ as well as the analysis of ancestry-informative SNPs.^207^ These methods are based on either two-, three-, or four-population comparisons, and are called, respectively, f2, f3, and f4.^151^ The f2-statistic determines the difference in allele frequencies between two populations. In comparison, f3 is a three-population test typically represented as f3(A,B; C) where each A, B, and C are a different population or individual. Depending on the configuration, it can be used to test if population C can be modeled as an admixture of the populations A and B or it can test for shared drift between populations A and B compared to the outgroup (C). In the case of admixture f3, the statistic is expected to be negative, while in the case of the outgroup f3 it is expected to be positive. Adding another population, an f4-statistic, similar to the D-statistic,^208^ which is also known as the ABBA-BABA statistic that has been developed to test for admixture in closely related populations, can be used to test for admixture and tree-ness using the formula f4(A,B; C,D) = (a-b)(c-d), where a, b, c, and d are allele frequencies in populations A, B, C, and D. When there is no additional admixture between A and B, and C and D, the statistic would be non-significant. As mentioned in the contamination section, f4 can also be used to identify presence of contamination within a dataset. The f-statistics calculation has been implemented in the software ADMIXTOOLS^151^ and an R-package *admixr*,^209^ as well as treemix,^210^ each of which have primers.

Another admixture modeling method qpAdm relies on the basic idea of the f4-statistic.^4^ The main difference lies in the ability of qpAdm to estimate the admixture proportions in the target population. The limitation of qpAdm is the need for the test of reference populations/individuals used as a tree scaffold to understand the relationship between the potential sources and the target.^211^ Choosing the outgroups correctly then becomes crucial for being able to disentangle how the source populations are related to the target. The use and limitations of qpAdm have been recently described.^211^

## Public databases

Both aDNA and forensic genetics use databases of modern populations to explore wider population genetics signatures and inform field-specific questions. In addition, in the aDNA field, the publication of genetic data from ancient individuals is commonplace. Databases are important resources that must be treated with care in relation to quality and accuracy of contributed data as well as considerations of privacy and respect for the individuals who have contributed their data. There have been vigorous discussions on best practices in both fields that include definitions of informed consent, acknowledgment of power dynamics, and potential implications of these databases on descendants and descendant communities, or genetic relatives of individuals in these databases.^88^^,^^89^^,^^212^^,^^213^^,^^214^^,^^215^^,^^216^

Within the forensics field, databases are typically used in three different ways. One is to provide references for determining haplogroup information for uniparental markers as exemplified by EMPOP (European DNA Profiling mtDNA Population Database)^217^ and YHRD (Y Chromosome Haplotype Reference Database).^218^ A second is to provide allele frequency information for different loci in order to aid in calculations for determining the certainty of an identification. These frequencies are also published for common forensic markers.^219^^,^^220^ The third is to provide searchable DNA profiles for identifying remains of missing people or providing leads to identify potential persons of interest in criminal cases. As of December 2022, EMPOP contains over 48,000 mitochondrial haplotypes that cover at least the hypervariable I region (over 4,200 complete mitochondrial genomes). This database has clear guidelines around nomenclature^221^ and quality control^217^^,^^222^ for the upload of new profiles. YHRD contains more than 350,000 Y-STR profiles and aims to provide accurate allele frequencies for Y chromosome STRs, although it contains Y-SNP data as well.^223^ Databases that provide autosomal frequency data vary by country and are typically divided based on either genetic ancestry or race, although there are ongoing debates as to the use (and accuracy) of these divisions.^224^

Laws and access to databases for identifying individuals vary by country, but are typically well defined and stringent.^225^ The inclusion of individuals in these databases also varies greatly and is an ongoing area of debate with criteria for inclusion ranging from citizenship,^226^ arrestee status, and category of criminal offense, and the biases of these databases due to racial and socioeconomic disparities.^227^^,^^228^ International database sharing among law enforcement also presents logistical and ethical questions, with international agencies such as INTERPOL creating protected measures for DNA profile searching.^229^^,^^230^ Outside of law enforcement, there are selected public databases of genetic data that law enforcement can search depending on the scope of the case in question. For example, the genetic profiles of the ∼1.4 million users of the genetic genealogical database GEDmatch are accessible for searching for missing person identifications if users make their profile public, but users can decide if their genetic data can be also used by law enforcement for searches related to violent crimes.^231^ However, there have been serious concerns that the profiles of GEDmatch users who opted out of sharing their genetic profiles with the law enforcement were still accessible to the police.^232^^,^^233^ Due to the ability of genetic genealogical analysis to identify deep family connections,^234^ investigators have estimated that a database similar to GEDmatch could be used to identify over 60% of individuals of European descent in the United States,^235^ raising significant concerns about privacy.

There are multiple publicly available databases and repositories that are commonly used to house aDNA data, such as: the European Nucleotide Archive (ENA) (https://www.ebi.ac.uk/ena/browser/), the Edmond Open Research Data Repository of the Max Planck Society (MPS) (https://edmond.mpdl.mpg.de/), Allen Ancient DNA Resources (AADR),^63^ the Poseidon database (http://www.poseidon-adna.org/#/), and others. AADR contains downloadable genotypes of ancient and present-day DNA data that cover various panels with the 1.2 million SNPs described in the “sampling and laboratory work” section. The AADR database also includes rich annotation information for each sample in the dataset, including the age of the sample, contamination metrics, coverage, sex, and geographic location, to name a few. The Edmond database can be used by MPS members to upload any files associated with their publications, including genotypes. Another resource is the Allen Ancient Genome Diversity Project/John Templeton Ancient DNA Atlas containing medium- and high-coverage shotgun sequencing data for 216 individuals (https://reich.hms.harvard.edu/ancient-genome-diversity-project). The authors ask the community to observe the Fort Lauderdale principles entitling the authors to be the first to present and publish the dataset. The Poseidon (http://www.poseidon-adna.org/#/) framework features a decentralized repository of genotyping and sequence data. Poseidon is managed through the efforts of the Department of Archaeogenetics of the Max Planck Institute for Evolutionary Anthropology. However, individual researchers are encouraged to submit packages of their genotyping data, with annotation files, and links to the BAM and FASTQ files. Finally, most aDNA studies published make the data available via the raw FASTQ files and/or BAM files aligned to the reference genome. ENA accession numbers can usually be found within the publication “Data Availability” statement.

## Study design

### Power analysis

One very important issue to consider when designing a study is the number of samples and/or individuals necessary to answer certain questions. This is relevant for both fields, as genetic analysis of skeletal remains typically requires destructive sampling.^236^ Different cultures and communities can have various points of view as to the implications of destruction of remains that should be weighed and discussed as part of a study’s initial design.^236^ In scenarios where destructive sampling is not a concern for cultural reasons, it can still result in the destruction of certain morphological features, such as teeth or petrous bone. Thus, a potential benefit of a large sample size has to be weighed against the consequences of this type of analysis. Power analysis is a way to assess the necessary sample size for certain research questions. For aDNA studies, this can refer either to the number of individuals, geographic locations, or number of loci covered. Demographic reconstructions,^237^ studies of natural selection,^238^ and single-locus analyses^239^ are examples of types of questions where a sufficient number of individuals and/or loci covered are necessary. For forensic casework related to HID, power analysis is still relevant for determining how much data are needed to reach conclusions around an identification. In addition, methodological and validation studies that seek to evaluate different steps within the wet and dry lab workflows should consider power analyses as part of the study design. It is also important to consider confounding factors, for example, how to isolate evaluations of the accuracy of genotyping and imputation methods. For methodological development and evaluation of bioinformatic tools, simulations are a powerful and necessary component.

## Summary and outlook

The development of new methods has enabled researchers to trace ancient populations and solve decades-old cold cases. Each year seems to bring a new study that pushes the limits as to what was previously thought possible for genetic research from degraded DNA. In addition, the number of aDNA labs and forensic genetic labs integrating DNA sequencing continues to increase, expanding the size of both fields and number of people performing these types of work. We hope that this review serves as both a primer for those new to working with sequencing data from degraded DNA and a marker for the current guidelines and limitations of different types of analyses. Due to these fluctuations, we encourage new and old members of the field to join and stay active in international and regional field-specific organizations such as the International Society for Biomolecular Anthropology, American Association of Anthropological Genetics, ISFG (which has language-specific working groups), SWGDAM, and ENSFI. In scenarios of no existing studies on the limitations of certain methods for working with incomplete data with signs of degradation and potential contamination commonly seen in forensics and aDNA fields, we also recommend performing simulations and/or downsampling tests in order to understand the power of any resulting associations from lower quality data. While this review has been limited to describing human genetic analysis from skeletal remains, these same methodological advancements have opened up new areas of study including ancient pathogens and sediment DNA that face similar (and added) challenges. As exploration into these new areas continues, we hope this review also serves to highlight the overlaps between forensics and ancient genetics and motivates future collaborations between these disciplines.

## Supporting citations

The following references appear in the supplemental information: ^56^^,^^57^^,^^58^^,^^91^^,^^92^^,^^93^^,^^94^^,^^95^^,^^97^^,^^98^^,^^101^^,^^102^^,^^103^^,^^104^^,^^108^^,^^110^^,^^111^^,^^112^^,^^113^^,^^142^^,^^143^^,^^144^^,^^145^^,^^147^^,^^151^^,^^164^^,^^165^^,^^166^^,^^178^^,^^180^^,^^191^^,^^193^^,^^197^^,^^198^^,^^199^^,^^209^^,^^210^^,^^240^^,^^241^^,^^242^^,^^243^^,^^244^^,^^245^^,^^246^^,^^247^^,^^248^^,^^249^^,^^250^^,^^251^^,^^252^^,^^253^.

## Supplemental information

Table S1. Non-exhaustive list of software commonly used in forensic and aDNA analysis
